# Value of TP53 Status for Predicting Response to Neoadjuvant Chemotherapy in Breast Cancer: A Meta-Analysis

**DOI:** 10.1371/journal.pone.0039655

**Published:** 2012-06-29

**Authors:** Min-Bin Chen, Ya-Qun Zhu, Jun-Ying Xu, Li-Qiang Wang, Chao-Ying Liu, Zhang-Yi Ji, Pei-Hua Lu

**Affiliations:** 1 Department of Medical Oncology, Kunshan First People’s Hospital Affiliated to Jiangsu University, Kunshan, People’s Republic of China; 2 Department of Radiotherapy and Oncology, The Second Affiliated Hospital of Soochow University, Suzhou, People’s Republic of China; 3 Department of Medical Oncology, Wuxi People’s Hospital Affiliated to Nanjing Medical University, Wuxi City, People’s Republic of China; 4 Department of General Surgery, Wuxi People’s Hospital Affiliated to Nanjing Medical University, Wuxi City, People’s Republic of China; National University of Ireland Galway, Ireland

## Abstract

**Background:**

Numerous studies have yielded inconclusive results regarding the relationship between tumor suppressor protein TP53 overexpression and/or *TP53* gene mutations and the response to neoadjuvant chemotherapy in patients with breast cancer. The purpose of the current study was therefore to evaluate the relationship between TP53 status and response to chemotherapy in breast cancer.

**Methods and Findings:**

A total of 26 previously published eligible studies including 3,476 cases were identified and included in this meta-analysis. TP53 status (over expression of TP53 protein and/or *TP53* gene mutations) was associated with good response in breast cancer patients who received neoadjuvant chemotherapy (total objective response: risk ratio [RR]  = 1.20, 95% confidence interval [CI]  = 1.09–1.33, p<0.001; pathological objective response: RR = 1.37, 95% CI = 1.20–1.57, p<0.01; total complete response: RR = 1.33, 95% CI = 1.15–1.53, p<0.001; pathological complete response: RR = 1.45, 95% CI = 1.25–1.68, p<0.001). In further stratified analyses, this association also existed among the studies using anthracycline-based neoadjuvant chemotherapy, and the association between response and the presence of gene alterations was stronger than that between response and immunohistochemistry positivity.

**Conclusion:**

The results of the present meta-analysis suggest that TP53 status is a predictive factor for response in breast cancer patients undergoing neoadjuvant chemotherapy. Further larger and well-designed prospective studies are required to evaluate the predictive role of TP53 status in clinical practice.

## Introduction

Neoadjuvant chemotherapy, also known as primary or induction chemotherapy, refers to chemotherapy administered before locoregional treatment, such as surgery and/or irradiation. Neoadjuvant chemotherapy has become the standard treatment for the management of locally advanced breast cancer, primarily because of its ability to downsize large tumors. Neoadjuvant chemotherapy is increasingly used for the treatment of early-stage breast cancer. However, despite generally high response rates, a small proportion of patients fail to respond to neoadjuvant chemotherapy, or even progress during therapy. Recent evidence suggests that biological markers may be useful for identifying those patients who would benefit from neoadjuvant chemotherapy [1].

The *TP53* gene is a prime candidate for predicting the response of tumors to classic chemotherapy [2]. It is a master gene in the stress response that plays a critical role in cancer development. *TP53* is the most frequently mutated gene in human cancer, with mutations occurring in at least 50% of human cancers [1]. It mediates checkpoint or stress responses to several insults and suppresses tumor formation through several mechanisms, including apoptosis, senescence, and autophagy [3]. Experimental evidence suggests a key role for TP53 in apoptosis in response to genotoxic agents [4], [5].

The use of TP53 status as a biological marker to predict the response of breast cancer to neoadjuvant chemotherapy, however, is disappointing, and the findings to date have shown conficting results [6]–[10]. Several studies [6], [9]–[11] found that patients with TP53 mutations often had better responses to therapy than those with normal TP53 status. Other studies [7], [8], [12], [13], however, evaluated TP53 status in breast cancer patients and drew different conclusions. The relevance of this gene to clinical therapy thus remains unknown. We therefore performed a meta-analysis of the value of TP53 status for predicting response to neoadjuvant chemotherapy in breast cancer.

## Materials and Methods

### Publication Search

PubMed, Embase, and Web of Science databases were searched (up to December 20, 2011) using the search terms: ‘TP53’, ‘p53’, ‘p53 protein’, ‘p53 mutation’, ‘17p13 gene’, ‘chemotherapy’ and ‘breast cancer’. All potentially eligible studies were retrieved and their bibliographies were carefully scanned to identify other eligible studies. Additional studies were identified by a hand search of the references cited in the original studies. When multiple studies of the same patient population were identified, we included the published report with the largest sample size. Only studies published in English were included in this meta-analysis.

### Inclusion and Exclusion Criteria

Studies included in this meta-analysis had to meet all of the following criteria: (a) evaluation of TP53 status for predicting the response to neoadjuvant chemotherapy in early-stage breast cancer, locally-advanced breast cancer, (b) described therapeutic response, (c) retrospective or prospective cohort study, (d) inclusion of sufficient data to allow the estimation of a risk ratio (RR) with 95% confidence intervals (95% CI), and (e) studies published in English. Letters to the editor, reviews, and articles published in books, or papers published in a language other than English were excluded.

### Data Extraction and Definitions

According to the inclusion criteria listed above, the following data were extracted for each study: the first author’s surname, publication year, country of origin, number of patients analyzed, types of measurement, and treatment. Data on the main outcomes were entered in tables showing the clinical and pathological responses to chemotherapy with respect to TP53 status. Information was carefully and independently extracted from all eligible publications by two of the authors (Chen and Zhu). Any disagreement between the researchers was resolved by discussions until a consensus was reached. If they failed to reach a consensus, a third investigator (Lu) was consulted to resolve the dispute.

We used the definitions and standardizations for ‘TP53’ and ‘response to chemotherapy’ as reported by Pakos et al. [14]. For consistency, we used ‘*TP53’* to denote the gene, ‘TP53’ for the expressed protein, and ‘TP53 status’ to refer to both the gene and protein markers. The correlation between protein and gene detection is not straightforward [9], [15]. *TP53* alterations increase the half-life of the TP53 protein, leading to nuclear accumulation of mutant *TP53*, which can be detected by immunohistochemistry (IHC). However TP53 protein accumulation measured by IHC does not necessarily correspond to *TP53* mutations. Thus, the overall analysis considered all studies, regardless of whether protein expression or gene mutation was being evaluated. Separate analyses for TP53 protein expression and *TP53* gene alterations were also performed. For studies using both protein and gene detection, we used the protein data but also examined the gene detection data, and found similar results (data not shown). TP53 status positive means patients with over expression of TP53 protein and/or *TP53* gene mutations. Response was defined as complete response (CR), partial response (PR), or objective response (OR) (OR  =  CR +PR). Non-response was defined as stable disease (SD) or progressive disease (PD), according to WHO criteria [16] or RECIST (Response Evaluation Criteria in Solid Tumors) criteria [17].

### Statistical Analysis

RR with 95% CIs was used to estimate the association between TP53 status and response to neoadjuvant chemotherapy in breast cancer patients. Subgroup analyses were performed to evaluate the effects of treatment regimens (anthracycline-based) and different methods of *TP53* gene determination (protein and gene). Heterogeneity assumption was checked using the Q test, and a p value >0.10 indicated a lack of heterogeneity among studies. The pooled RR was calculated using a fixed-effects model (the Mantel–Haenszel method) or a random-effects model (the DerSimonian and Laird method), according to the heterogeneity. Funnel plots and the Egger’s test were employed to estimate the possible publication bias. We also performed sensitivity analysis by omitting each study or specific studies to find potential outliers. Statistical analyses were conducted using Stata (version SE/10; StataCorp, College Station, TX). p values for all comparisons were two-tailed and statistical significance was defined as p<0.05 for all tests, except those for heterogeneity.

## Results

### Eligible Studies

A total of 1,223 articles were retrieved by a literature search of the PubMed, Embase, and Web of Science databases, using different combinations of key terms. As indicated in the search flow diagram (Figure S1), 26 studies reported at least one of the outcomes of interest and were finally included in the meta-analysis [2], [6]–[13], [15], [18]–[33]. The characteristics of the eligible studies are summarized in Table 1. Twenty-one of the studies employed IHC, eight employed gene detection (including genomic sequencing, DNA microarray, Functional Analysis of Separated Allele in Yeast [FASAY]), two employed both methods and one employed three methods (Table 1). The sample sizes in all the eligible studies ranged from 20–1,469 patients (median  = 73 patients, mean  = 134 patients, standard deviation [SD]  = 54). Overall, the eligible studies included 3,476 patients. Eighteen of the studies were conducted in European or North American populations with mixed but mostly white participants (1,460 patients), whereas eight were conducted in East Asian populations (748 patients).

**Table 1:** Characteristics of studies included in the meta-analysis.

Author	Year	Country	Cases	TreatmentSubgroup of treatment		Detection	Response	
Makris et al. [18]	1997	UK	80	mitoxantrone, methotrexate (± mitomycin C) and tamoxifen	N	IHC	clinical response	CR + PR
Kandioler-Eckersberger et al. [9]	2000	Austria	67	FEC or paclitaxel	N	PCR amplification sequencing and IHC	clinical response	CR + PR
Geisler et al. [20]	2001	Norway	90	weekly doxorubicin scheduled for 16 weeks	A-b	IHC, TTGE and sequencing	clinical response	PR
Schneider et al. [19]	2001	Spain	52	FAC or CMF	N	IHC	clinical response	CR + PR
Aas et al. [11]	2003	Norway	90	doxorubicin	A-b	IHC	clinical response	PR+SD
Anelli et al. [6]	2003	Brazil	73	AT	A-b	IHC	clinical response	CR + PR
Bonnefoi et al. [22]	2003	Switzerland	179	FEC,EC + G-CSF	A-b	IHC	clinical response	CR
Martin-RIHCard et al.[]	2003	Spain	38	FAC or FEC	A-b	IHC	clinical response	CR + PR
Geisler et al. [21]	2003	Norway	35	FUMI regimen	N	IHC	clinical response	PR
Mathieu et al. [24]	2004	France	129	AVCMF or FAC/FEC	A-b	IHC	Pathologic response	CR
Deissler et al. [23]	2004	Germany	50	anthracycline/taxane	A-b	FASAY	clinical response	CR
Kim et al. [26]	2005	Japan	63	docetaxel	N	IHC	Pathologic response	RR
Learn et al. [25]	2005	USA	121	AC vs. AC+D	A-b	IHC	Pathologic response	CR
Bertheau et al. [29]	2007	France	80	EC	A-b	FASAY	Pathologic response	CR
Tiezzi et al. [8]	2007	Brazil	60	CMF or FEC	N	IHC	clinical response	CR + PR
Keam et al. [27]	2007	Korea	145	docetaxel and doxorubicin	A-b	IHC	Pathologic response	CR + PR
Lee et al. [28]	2008	Korea	61	AT	A-b	IHC	clinical response	RR
							Pathologic response	CR
Zhou et al. [7]	2008	China	135	taxanes and anthracycline	A-b	IHC	Pathologic response	CR
Yonemori et al. [12]	2009	Japan	44	trastuzumab-containing neoadjuvant	N	IHC	Pathologic response	CR
Shekhar et al. [30]	2009	USA	20	AC, AT, FAC, FAT	A-b	IHC	clinical and pathologic response	CR + PR
Silver et al. [32]	2010	USA	22	DDP	N	IHC	clinical and pathologic response	CR + PR
Masuda et al. [31]	2010	Japan	33	FEC100 and taxanes	A-b	IHC	Pathologic response	CR
Sanchez-Munoz et al. [10]	2010	Spain	73	EC followed by GP (+ trastuzumab in Her2 patients)	A-b	IHC	Pathologic response	CR
Bonnefoi et al. [2]	2011	Europe	1469	FEC VS. T-ET	A-b	FASAY	clinical and pathologic response	CR
Ono et al. [33]	2011	Japan	179	anthracycline-based regimens	A-b	IHC	Pathologic response	CR
Oshima et al. [15]	2011	Japan	88	P-FEC	A-b	genomic sequencing, DNA microarray and IHC	Pathologic response	CR

IHC, immunohistochemistry; FEC, 5-fluorouracil, epirubicin, and cyclophosphamide; FAC, 5-fluorouracil, doxorubicin, and cyclophosphamide; CMF, cyclophosphamide, mitomycin C and 5-fluorouracil; AVCMF, doxorubicin, vincristine, cyclophosphamide, methotrexate and 5-fluorouracil; P-FEC, sequential paclitaxel and 5-FU/epirubicin/cyclophosphamide; FUMI regimen, 5-fluorouracil (1,000 mg/m^2^ on days 1 and 2) and mitomycin; EC, epirubicin and cyclophosphamide; A, doxorubicin; E, epirubicin; T, docetaxel; P, paclitaxel; G, gemcitabine; FASAY, RNA-based functional assay in yeast; TTGE, temporal temperature gradient gel electrophoresis. N, can not be grouped; A-b, anthracycline-based neoadjuvant chemotherapy.

### Correlation of TP53 Status with Response to Neoadjuvant Chemotherapy in Breast Cancer Patients

Among the studies of breast cancer patients who received neoadjuvant therapy, 26 studies involving 3,476 patients contributed data on total OR (clinical OR + pathological OR). TP53 status-positivity was significantly associated with improved total OR among patients treated with neoadjuvant therapy (RR = 1.20; 95% CI = 1.09–1.33; p<0.001, Figure S2). Thirteen studies involving 2,761 patients contributed data on pathological OR. TP53 status-positivity was significantly associated with improved pathological response (RR = 1.37; 95% CI = 1.20–1.57; p<0.001). Fifteen studies involving 2,736 patients contributed data on total CR. TP53 status-positivity was significantly associated with improved total CR (RR = 1.33; 95% CI = 1.15–1.53; p<0.001). Finally, 12 studies involving 2,434 patients provided information on pathological CR. TP53 status-positivity was significantly associated with significant improvements in pathological CR (RR = 1.45; 95% CI = 1.25–1.68; p<0.001, Figure S3). For studies using both clinical and pathological responses, we used the pathological-response data, but also examined the clinical-response data and found similar results (data not shown).

### Subgroup Analysis

Among the 26 studies in the neoadjuvant subgroup, 18 used anthracycline-based neoadjuvant chemotherapy,while the remaining studies can not be grouped (table 1). The results of the anthracycline-based neoadjuvant chemotherapies were therefore calculated. TP53 status-positivity was associated with improved response in breast cancer patients who received anthracycline-based neoadjuvant chemotherapy (total OR: RR = 1.18, 95% CI = 1.04–1.33, p = 0.010, Figure S4; pathological OR: RR = 1.33, 95% CI = 1.19–1.62, p = 0.005; total CR: RR = 1.33, 95% CI = 1.15–1.54, p<0.001; pathological CR: RR = 1.45, 95% CI = 1.24–1.69, p<0.001). For studies using both clinical and pathological responses, we used the pathological-response data, but also examined the clinical-response data, and similar results were obtained (data not shown).

Different measurements of TP53 status (either by protein or gene detection) have been used to evaluate associations with favorable responses to neoadjuvant chemotherapy. We therefore calculated the associations using both protein and gene statuses of TP53. The results of subgroup analysis are presented in Table 2. For gene detection, TP53 status-positivity was significantly associated with increased total OR (RR = 1.41, 95% CI = 1.20–1.65, p<0.001, Figure S5), total pathological response (pathological response: RR = 1.49; 95% CI = 1.24–1.79; p<0.001), total CR (RR = 1.46; 95% CI = 1.22–1.75; p<0.001) and pathological CR (RR = 1.49; 95% CI = 1.24–1.79; p<0.001) among patients treated with neoadjuvant chemotherapy. For protein-based detection, TP53 status-positivity was significantly associated with increased total OR (RR = 1.22; 95% CI = 1.01–1.48; p = 0.041) and total CR (RR = 1.32; 95% CI = 1.02–1.69; p = 0.032) among patients treated with neoadjuvant chemotherapy, but not with total OR (RR = 1.06; 95% CI = 0.94–1.20; p = 0.310) or total CR (RR = 1.15; 95% CI = 0.92–1.43; p = 0.235). For studies using both clinical and pathological responses, we used the pathological-response data, but also examined the clinical response data, and the results were similar (data not shown).

**Table 2:** Risk ratio for the association between TP53 status and response to neoadjuvant chemotherapy.

Comparison	Total OR*				Pathological OR				Total CR*				Pathological CR			
	N	RR (95%CI)	p value	Ph	N	RR (95%CI)	p value	Ph	N	RR (95%CI)	p value	Ph	N	RR (95%CI)	p value	Ph
All studies	26	1.20 (1.09–1.33)	<0.001	0.292	15	1.37 (1.20–1.57)	<0.001	0.329	15	1.33 (1.15–1.53)	<0.001	0.095	12	1.45 (1.25–1.68)	<0.001	0.391
Treatment																
Anthracycline-based	17	1.18 (1.04–1.33)	0.010	0.298	10	1.33 (1.19–1.62)	0.005	0.109	12	1.33 (1.15–1.54)	<0.001	0.031	9	1.45 (1.24–1.69)	<0.001	0.175
Type of measurement																
Protein	21	1.06 (0.94–1.20)	0.310	0.796	12	1.22 (1.01–1.48)	0.041	0.637	12	1.15 (0.92–1.43)	0.235	0.209	9	1.32 (1.02–1.69)	0.032	0.659
Gene	8	1.41 (1.20–1.65)	<0.001	0.207	4	1.49 (1.24–1.79)	<0.001	0.089	5	1.46 (1.22–1.75)	<0.001	0.076	4	1.49 (1.24–1.79)	<0.001	0.089

Subgroup analysis was performed when there were at least two studies in each subgroup.

N, number of studies; Ph, p value of Q-test for heterogeneity.

* For studies using both clinical and pathological responses, we used the pathological response data, but also examined the clinical response data, and found similar results (data not shown).

#One study (Oshima et al. [15]) used both genomic sequencing and DNA microarray analysis for gene measurement; we used genomic sequencing data, but also also examined the DNA microarray data, and found similar results (data not shown).

### Publication Bias

Begg’s funnel plot and Egger’s test were used to estimate the publication bias of the included literature. The shapes of the funnel plots showed no evidence of obvious asymmetry(Figure S6), and Egger’s test indicated the absence of publication bias (p>0.05). Moreover, sensitivity analysis was carried out to assess the influence of individual studies on the summary effect. Trastuzumab would likely have increased the chances of response when combined with a taxane in two of the studies which included patients with HER-2 positive disease, there may be some discordance in response rates in the newer studies compared to the older studies prior to the advent of trastuzumab, this may have falsely credited the anthracycline for the benefit seen and introduced confounding. However, the corresponding pooled RRs were not substantially altered whether or not these studies were included. No individual study dominated this meta-analysis, and the removal of any single study had no significant effect on the overall results (total OR: RR ranged from 1.12[95% CI = 1.01–1.25] to 1.22 [95% CI = 1.10–1.35]; pathological OR: RR ranged from 1.42 [95% CI = 1.22–1.66] to 1.51 [95% CI = 1.18–1.93]; total CR: RR ranged from 1.24 [95% CI = 1.01–1.56] to 1.37 [95% CI = 1.18–1.59]; pathological CR: RR ranged from 1.42 [95% CI = 1.22–1.66] to 1.47 [95% CI = 1.26–1.76]).

## Discussion

TP53 status had been shown to play a pivotal role in the response to a large panel of anticancer drugs. Previous studies suggested that breast cancers with *TP53* mutations might be either resistant or sensitive to anticancer drugs. However, the issue could not be resolved, because most of the available clinical reports involved small sample sizes, and the results were therefore unable to determine the value of TP53 status for predicting the response to chemotherapy. Additionally, IHC, which lacks sensitivity and specificity, or various DNA sequencing techniques, some of which also lack sensitivity, were the main techniques used in these studies. We therefore concluded that a meta-analysis was the best way of evaluating the association between TP53 status and response to neoadjuvant chemotherapy in a large population.

The current meta-analysis of 26 studies systematically evaluated the association between TP53 status and response to neoadjuvant chemotherapy in a large population. The results indicate that altered TP53 status may predict good response rates to neoadjuvant chemotherapy in patients with breast cancer. TP53 status was associated with total and pathologically relevant increases in OR and CR. Stratification according to different treatments showed that altered TP53 status was significantly associated with increased OR and CR in patients who received anthracycline-based neoadjuvant chemotherapy. Further stratification by gene detection revealed imprecise results, but amplification of the *TP53* gene was also associated with relevant increases in OR and CR (both total and pathological); however, although overexpression of TP53 was associated with relevant increases in pathological OR and CR, it was not associated with total OR and CR. Gene detection was associated with advantages regarding response rates to neoadjuvant chemotherapy in patients with breast cancer. Gene detection may thus be a useful approach in future prospective studies.

Despite our attempts to perform a comprehensive analysis, there were some limitations associated with this meta-analysis. First, the meta-analysis may have been influenced by publication bias, we limited the search to studies performed in English, and we did not search conference proceedings and abstract books, which may have introduced publication bias to meta-analysis. We tried to identify all relevant data and retrieve additional unpublished information, some missing data were unavoidable. Second, the studies used different measurements of TP53 status (either protein or gene detection), and the cut-off values for TP53 for overexpression by IHC and for gene amplification differed between studies. Standardization is therefore of great importance for obtaining an accurate assessment of the clinical significance of TP53 status. Although we made considerable efforts to standardize definitions, some variability in definitions of methods, measurements, and outcomes among studies was inevitable. Third, our analysis was observational in nature, and we therefore cannot exclude confounding as a potential explanation of the observed results. Despite these limitations, this meta-analysis had several strengths. First, a substantial number of cases were pooled from different studies, and 3,476 subjects represent a sizeable number, significantly increasing the statistical power of the analysis. Secondly, no publication biases were detected, indicating that the pooled results may be unbiased.

This study is the first meta-analysis to assess the usefulness of TP53 status for predicting the response of breast cancer patients to neoadjuvant chemotherapy. Our data support TP53 status as a useful predictive factor for assessing treatment response to neoadjuvant chemotherapy in breast cancer patients. However, future prospective studies with large sample sizes and better study designs are required to confirm our findings. Moreover, the interactions of this marker with other molecular markers such as HER-2 [34] or estrogen receptor [35] remain unknown, and should be topics for further investigation.

## Supporting Information

Figure S1 **Improving the quality of reports of meta-analyses of randomized controlled trials; the Quality of Reporting of Meta-Analyses (QUOROM) statement flow diagram.**
(EPS)

Figure S2 **Forest plots of RR were assessed for association between TP53 and total OR among breast cancer patients treated with neoadjuvant therapy.**
(EPS)

Figure S3 **Forest plots of RR were assessed for association between TP53 and pathological CR among breast cancer patients treated with neoadjuvant therapy.**
(EPS)

Figure S4 **Forest plots of RR were assessed for the evaluation of total OR in anthracycline-based settings.**
(EPS)

Figure S5 **Forest plots of RR were assessed for the evaluation of total OR in gene-based detection settings.**
(EPS)

Figure S6 **The funnel plot shows that there was no obvious indication of publication bias for the outcome of total OR.**
(EPS)
